# Supplementary material for: Correlation between the tissue ghrelin presence, disease activity and laboratory parameters in ulcerative colitis patients; immunohistochemical study
Source: PLoS One. 2022 Nov 9;17(11):e0276065. doi: 10.1371/journal.pone.0276065 (PMC9645640; doi:10.1371/journal.pone.0276065)
Supplement: Attachment — Submitted filename: response ghrelin yyy.doc [file pone.0276065.s002.doc]

3.07.2022

Dear Editor,

We would like to thank you for considering our manuscript entitled “ **Correlation between the tissue ghrelin presence, disease activity and laboratory parameters in ulcerative colitis patients: An immunohistochemical study**

” (**Manuscript No:** PONE-D-22-04274

**)** for publication in the PLOS ONE. We also would like to thank to the Editorial board and the Referees for their important contributions, which finally improved our paper.

We have carefully reviewed the comments of the Reviewers and revised the manuscript accordingly. Below please find the answers to the Reviewers’ comments and the revised version of our paper. Please do not hesitate to make any further change in the text according to the requirements of the journal.

Yours faithfully,

Memduh Sahin, MD

**Corresponding author:**

Memduh Sahin M.D.

Basaksehir Cam and Sakura City Hospital,

**Basaksehir/ İSTANBUL**

E-mail:memsahinsahin@otmail.com

**The following answers were given and the revisions made according to the Reviewers’ comments:**

***Reviewer #1***

**Comment 1:**

“First of all, the authors check correctness of description of groups of people participating in the study and the presented results. In the abstract, Materials and Methods, and Table 2, the authors stated that group A contains the patients with ulcerative colitis without the presence of ghrelin staining in biopsy samples; whereas patients with ulcerative colitis and presence of ghrelin staining in biopsy samples were gathered in group B. In addition, the authors stated that Mayo score/disease activity index (DAI) is patients with ulcerative colitis was significantly higher in group A than group B (abstract and Table 2). Final conclusions at the end of the manuscript are in agreement with mentioned above data. On the other hand, the authors present opposite conclusion in the abstract that “Colonic ghrelin staining in UC patients seems to be associated with the increased activity of this disease”. Moreover, the authors stated that “DAI were found to be higher in UC patients with showing ghrelin positivity (Group A) (Discussion, paragraph 3). Inconsistences in the conclusions should be checked and corrected.
”

**Answer 1:**

“We would like to express our gratitude to the Reviewer for this kind appreciation of our work and the outstanding criticisms, which finally improved our paper.’’

In our study, ulcerative colitis disease activity was found to be lower in cases with ghrelin staining. The statements in the abstract and discussion part of the research were corrected with these sentences.

Abstract“. Mayo score/disease activity index (DAI) for UC was significantly higher in Group A compared to Group B (p=0.03).

There were no differences in the amount of colonic ghrelin staining between healthy individuals and UC patients. Colonic ghrelin staining in UC patients seemed to be associated with the decreased activity of this disease.’’

Discussionc ‘ ‘In our study, higher levels of staining were found in the control group in terms of tissue staining amounts, but this data was not statistically significant. DAI was found to be higher in UC patients with negative ghrelin (Group A).’’

Conclusion’’Disease activity detected in the group with positive ghrelin staining, was found to be statistically lower than the negative stained group’’

**Comment 2:**

“All abbreviations should be presented in full form at the place where they are used for the first time in the abstract and repeated in the main body of the manuscript.”

**Answer and Revision 2:**

Abbreviations in the article are repeated in the same way as they were first mentioned in the whole article. “” .

**Comment 3:**

“. Introduction. The authors should write that in the gut, the protective and/or therapeutic effect of ghrelin was demonstrated in different organs (PMID: 34638910), including among others, in the oral cavity (PMID: 24304579; PMID: 29151078), pancreas (PMID: 14726611; PMID: 17084100; PMID: 25594510), stomach (PMID: 24622834) and duodenum (PMID: 19439811).
"

**Answer and Revision 3:** The introduction part has been changed by adding the expressions suggested by the referees. Sources of information on these expressions are also included. “Protective and/or therapeutic effect of ghrelin has been demonstrated in different organs (17), including among others, the oral cavity (18; 19), pancreas (20; 21; 22), stomach (23) and duodenum (24).’’

**Comment 4:** The authors should write that previous studies showed that ghrelin exhibit the beneficial effects in different experimental models of colitis. Among others, the protective and/or therapeutic effect of ghrelin was observed in experimental colitis evoked by acetic acid (PMID: 26769837; PMID: 27598133), dextran sodium sulfate (PMID: 26713317)

**Answer and Revision 4:** The introduction part has also been changed by adding the expressions suggested by the referees. Sources of information on these expressions are also included. “Previous studies have shown that ghrelin exhibited the beneficial effects in various experimental models of colitis. Among others, protective and/or therapeutic activity of ghrelin has been observed in experimental colitis induced by acetic acid (25; 26) and dextran sodium sulfate (27).’’

**Comment 5:** Major revision of English is necessary.

**Answer and Revision 5:** The article has been re-evaluated in terms of English, and the detected English spelling errors and sentence styles have been changed and corrected.

***Reviewer #2***

***Comment 1:*** : It is not clear how ghrelin is implicated in UC pathogenesis. Why authors choose only UCand no CD patients. Did the patients received biological agents? It is very simple approach to have only immunohistochemistry data. What can be the relation of grelin expression and biological therapies

**Answer and Revision 1:** In our study, only ulcerative colitis patients were included in order to make the group homogeneous. A study with Crohn's patients or a comparison of ulcerative colitis and crohn's patient group may be suggestive for another study.

*Since only two (2 infliximab treatment patient)* patients received biological treatment in the study, statistical evaluation could not be made for this. Our results can be instructive for the comparison of patients who received and did not receive biological treatment. Examination of patients receiving biologic agents may be the subject of another study. The explanations about the mentioned questions and the treatments received by the patients are explained in the discussion section. “In order to ensure homogeneity, only ulcerative colitis patients were included in the study. Studies on Crohn's disease patients may be a prompt for another study. Another limitation in our study was the scarce use of biologic agents in the patient group (only 2 patients were receiving infliximab). Therefore, in the study, treatment with biological agents was evaluated among patients receiving immunosuppressive therapy, and this group includes patients receiving steroid, azathioprine, and infliximab. There were no patients receiving adalimumab treatment in the study.’’

The treatments received by the patients are summarized in Table 4. Immunosuppressive and biological treatments taken by the patients were added to this table in more detail and revision was applied.

**Journal Requirements:** -During your revisions, please note that a simple title correction is required: 'Relationship' is misspelled. Please ensure this is updated in the manuscript file and the online submission information

- Thank you for stating the following financial disclosure: "No Fınancial Support"

At this time, please address the following queries

a) Please clarify the sources of funding (financial or material support) for your study. List the grants or organizations that supported your study, including funding received from your institution. 
b) State what role the funders took in the study. If the funders had no role in your study, please state: “The funders had no role in study design, data collection and analysis, decision to publish, or preparation of the manuscript

**Answer and Revision: :** The title of the article has been changed to "Correlation between the tissue ghrelin presence, disease activity and laboratory parameters in ulcerative colitis patients: An immunohistochemical study’’

No financial support was received in the study, and the research costs were covered by the researchers' own budget and means.
